# Behavior without beliefs: Profiles of heteronormativity and well-being among heterosexual and non-heterosexual university students in Chile

**DOI:** 10.3389/fpsyg.2022.988054

**Published:** 2022-08-15

**Authors:** Ligia Orellana, Tatiana Alarcón, Berta Schnettler

**Affiliations:** ^1^Núcleo de Ciencias Sociales y Humanidades, Universidad de La Frontera, Temuco, Chile; ^2^Núcleo Científico Tecnológico en Biorecursos (BIOREN-UFRO), Universidad de La Frontera, Temuco, Chile; ^3^Facultad de Ciencias Agropecuarias y Forestales, Universidad de La Frontera, Temuco, Chile; ^4^Facultad de Especialidades Empresariales, Universidad Católica de Santiago de Guayaquil, Guayaquil, Ecuador

**Keywords:** Heteronormativity, subjective well-being, university students, latent profile analysis, LGBTQI+

## Abstract

Heteronormativity comprises essentialist, binary beliefs about sex and gender, and normative behaviors derived from those beliefs. There is scarce literature on how heteronormative attitudes and well-being variables are concurrent among individuals who are heterosexual or gay, lesbian, bisexual, and of other queer sexual identities (LGBQ). The objective of this study was to distinguish profiles of university students based on essentialism and normative behavior, two dimensions of heteronormativity, and to characterize these groups by sexual orientation and gender, perceived social support, physical and mental health, and life satisfaction. A sample of 552 university students in Temuco, Chile, responded to an online questionnaire consisting of sociodemographic questions, the Scale of Heteronormative Attitudes and Beliefs, the Life Satisfaction Scale, the Health-Related Quality of Life Index, and the Multidimensional Scale of Perceived Social Support. We used Latent profile analysis to distinguish profiles based on significant score differences in Essentialism and Normative behavior. We identified four heteronormativity profiles: *High heteronormativity* (34.85%), with a significant proportion of heterosexuals and men; *Low heteronormativity* (25.59%), comprising a significant proportion of students who were non-binary, and LGBQ; *Heteronormativity focused on normative behavior* (20.42%), with a significant proportion students who were men or non-binary, and who were lesbian, gay or bisexual or preferred not to disclose their sexual orientation; and *Heteronormativity focused on essentialism* (19.14%), with a significant proportion of heterosexuals and women, and individuals who preferred not to disclose their sexual orientation. The four profiles differed in the proportions of students by faculty and area of residence (urban/rural), and by life satisfaction, self-perceived mental health, and perceived social support. These results show that patterns of association between heteronormativity and subjective well-being are heterogeneous among heterosexual and non-heterosexual individuals. Some of these patterns may respond to the COVID-19 pandemic, which has disrupted daily life and social dynamics. These findings expand our understanding of advantageous and disadvantageous conditions associated with maintaining heteronormativity attitudes, particularly among non-heterosexual individuals.

## Introduction

Psychological research increasingly recognizes sexual orientation as an attribute that configures the individuals’ personal and social life. Powdthavee and Wooden (2015) have shown that sexual orientation is one of many determinants of life satisfaction and of intermediate variables, such as health, employment, and social support. Other studies on well-being and sexual orientation consistently report that lesbian, gay, bisexual, and other queer individuals such as asexual and pansexual (LGBQ) are at a higher risk of experiencing poorer health and well-being outcomes than heterosexuals (Meyer, 2003; Powdthavee and Wooden, 2015; Cooke, 2018; Pachankis and Bränström, 2018; Mann et al., 2019; Perales, 2019). These disparities are explained by the minority stress model (Meyer, 2003; Meyer et al., 2021), which posits that LGBQ people (often including transgender, non-binary, and intersex individuals, LGBTQI+), as members of a marginalized group, face additional stressors than the general population. For instance, the COVID-19 pandemic and the measures enforced to contain it have imposed severe stressors for the general population, affecting their mental and well-being (Orellana and Orellana, 2020; Barrientos et al., 2021). These effects, however, can be even more pronounced among LGBTQI+ people as they experience more social isolation and more difficulties in expressing their identities (Barrientos et al., 2021), among other conditions.

The origin of minority stressors, and the overall stigmatization of LGBQ people have been traced to the assumption that heterosexual orientation and a binary gender identity are “normal” (Mann et al., 2019), while non-heterosexual and non-binary gender identities are deviant. Heteronormativity is built upon these assumptions (Habarth, 2014; Phipps, 2020). The “normal, acceptable sexual behavior” is heterosexuality, the attraction to persons of another gender assuming that there are two “opposite” genders (Seal, 2019). On this basis, Habarth (2014) defines heteronormativity as the reinforcement of heterosexuality as normal and natural, and as the standard to define what is acceptable for gender roles, sexual behavior, and gender and sexual identities and relations. Heteronormativity does not admit fluidity, only deviations from the norms, being bound with binary notions of sex (male/female), gender identity (man/woman), gender roles (masculine/feminine), and, more recently, sexual identity (straight/gay, but “only a certain kind of homosexuality,” Seal, 2019, p. 28).

Heteronormativity entails cultural norms (Farvid, 2015; Bible, 2020), reflected and reinforced in social institutions and structures, such as healthcare (Enson, 2015; Vergara, 2020), education (Enson, 2015), and the workplace (Corlett et al., 2022). This construct, however, also includes cognitive processes that form the basis of prejudice, victimization, and discrimination toward LGBTQI+ people (Habarth, 2014; Ray and Parkhill, 2021; Corlett et al., 2022). Habarth (2014) thus proposed that heteronormativity comprises two dimensions: Essentialist beliefs about the binary nature of sex and gender (Essentialism), and attitudes derived from these beliefs regarding expected behaviors of people as man or woman, individually and in relationships (Normative behavior). This two-dimensional structure of heteronormativity is supported by psychometric evidence from Italy (Scandurra et al., 2021) and Chile (Alarcón et al., manuscript under review). Other research has linked heteronormativity to personality traits (e.g., openness to experience), political attitudes (e.g., right-wing authoritarianism), sexual prejudice, and demographic variables such as sexual orientation and gender (Habarth, 2014; Habarth et al., 2019a,b; Ray and Parkhill, 2021; Scandurra et al., 2021). Further explorations of heteronormativity posit that its nature transcends the realm of sexuality and gender and involve other identity markers and life conditions such as family structure, socioeconomic status, and ethnic origin (Seal, 2019; Pollitt et al., 2021).

Heteronormativity negatively affects all people (Seal, 2019), because it relates to power and hierarchical relations, idealization of specific types of relationships and families, rigid gender norms and stereotypes, normalization of sexual coercion, among others (Farvid, 2015; Wilson, 2022). Nevertheless, the effects of heteronormativity can be more pronounced on queer populations (McDermott et al., 2021). To date, most empirical research on heteronormativity follows a variable-centered approach, that is, observing the average effect of this construct on all individuals in a sample (Bouckenooghe et al., 2018). In our study, we proposed a person-centered approach to group individuals, distinguishing profiles based on how heteronormativity and well-being variables manifested and associated with one another (Choi et al., 2019; Withers, 2020). We thus used Latent Profile Analysis (LPA) to examine groups of individuals or profiles based on Habarth’s (2014) two dimensions of heteronormativity, Essentialism and Normative behavior. LPA allows to explore heterogeneity in a population, showing individual differences in psychological phenomena and using supporting variables associated with the latent group membership (e.g., Hardy, 2019). Using LPA, we sought to examine the distinct configurations of Essentialism and Normative behavior and their associations with well-being variables.

The population of interest in this study is university students, using the emerging adulthood framework that covers the developmental period from ages 18 to 29 (Arnett, 2000, 2014; Nelson, 2020). The literature on emerging adulthood characterizes this period as heterogeneous, inasmuch the individuals’ choices prevail over the timed accomplishment of developmental milestones compared to previous generations, such as leaving the parental home, getting married, and having children (Nelson, 2020). Researchers have also characterized emergent adulthood as a period in which “nothing is normative”; it is a period of stress and instability, but it also affords individuals opportunities for exploration, reorganization of relationships and self-focus, and ultimately, for establishing their life trajectory (Nelson, 2020). Of interest to our study, emergent adulthood is also marked by an exploration of sexual identity (Arnett, 2007; Hong et al., 2015). For LGBTQI+ emergent adults, attending university entails new conditions and resources that allow them to disclose their sexual orientation or gender identity in their expanding social environment (Nelson, 2020). Studies with university students in Chile have explored both their experiences as emergent adults (Barrera-Herrera and Vinet, 2017), and their well-being and life satisfaction (Schnettler et al., 2015). Findings from these studies highlight that, compared to populations in other developmental periods, university students experience a distinct development of sexual identity and social relationships (Barrera-Herrera and Vinet, 2017), and report lower life satisfaction (Schnettler et al., 2015). Furthermore, the COVID-19 pandemic has disrupted the life trajectories of emergent adults, altering their access to education and sources of social support outside the home, and often confining them to the family home (Orellana and Orellana, 2020; Barrientos et al., 2021). For LGBTQI+ people, these alterations can also mean having to conceal their sexual or gender identity or expressing it while dealing with their family’s rejection (Barrientos et al., 2021).

The context of higher education tends to be more welcoming toward sexual and gender identities than other social spheres, but it still reinforces a heteronormative worldview (Rodríguez-Mena et al., 2018; Bautista, 2019; Seal, 2019). Research on heteronormativity in higher education (Hong et al., 2015; Seal, 2019) reveals an environment of direct and structural discrimination, including language, administrative practices, heteronormative examples in class, relations between students and staff, and discussions that intend to tackle discrimination but reinforce the othering of LGBTQI+ people. Heteronormativity manifests not only in the classroom, but in social spaces in campus, such as cafeterias, soccer fields, hallways, and bathrooms (Maldonado-Ramírez, 2015; Seal, 2019). Heteronormativity can also be displayed differentially in masculinized and feminized fields (Maldonado-Ramírez, 2015; Corlett et al., 2022). Of note in this regard, Habarth et al. (2019a) have shown that attaining higher education is associated with lower heteronormativity in women, but not in men, suggesting that attending university on its own does not counter heteronormative attitudes. Universities also tend to be in urban areas, which have been associated with the free development and expression of sexual identity (Barrientos-Delgado et al., 2014; Giano et al., 2020). Giano et al. (2020) indicate that most studies with LGBTQI+ populations are conducted in urban areas, although these areas in conservative regions can still be characterized by opposition to these non-normative identities. On the other hand, according to the above authors, rural areas are not homogeneous, and they may also present protective factors for LGBTQI+ populations.

Heteronormativity varies by gender and sexual orientation. There is evidence that heteronormativity is higher in men than in women, higher in heterosexuals than in non-heterosexuals, and higher in gay men and lesbians than in bisexuals (Habarth, 2014; Habarth et al., 2019a,b). In terms of gender, heteronormativity maintains a social hierarchy, and Ray and Parkhill (2021) state that heterosexual men who adhere more to heteronormativity feel more threatened in their social status by gay men. In terms of sexual orientation, Pollitt et al. (2021) indicate that there is scarce research on how LGBTQI+ young adults navigate heteronormativity, but evidence shows that they can both challenge and reinforce gender expression norms. Discourses may present LGBTQI+ identities and heteronormativity as mutually excluding (see Beltrán y Puga, 2012), but heteronormativity is engrained in daily life and it affords benefits to those who endorse these attitudes, even if they belong to socially disadvantaged groups, such as women and non-heterosexual people (Habarth et al., 2019b; Seal, 2019). For instance, Pollitt et al. (2021) found that LGBQ young adults have internalized the traditional “true” family formation (blood relations and children born from biological parents) as an ideal, even if it appears unattainable to them.

The link between heteronormativity and subjective well-being is an emergent interest in the literature. Researchers have reported differences in life satisfaction based on sexual orientation (Powdthavee and Wooden, 2015; Pachankis and Bränström, 2018; Habarth et al., 2019b; Bartram, 2021), and these differences may be partly explained by heteronormativity (Mann et al., 2019). Life satisfaction is the cognitive component of subjective well-being, and it is a measure of the person’s assessment of their overall life conditions (Diener et al., 1985). In our study, we follow the bottom-up perspective of life satisfaction, which assumes that individuals’ life satisfaction depends on their satisfaction in concrete areas or domains of their life (Loewe et al., 2014). The distinctions in life satisfaction and other well-being factors by sexual orientation are nuanced, however, as these have been observed between heterosexuals and non-heterosexuals, but also within the latter group, and with distinctions by gender and other sociodemographic characteristics. In Chile, Barrientos et al. (2017) found that lesbian women reported higher life satisfaction than gay men, as the latter experience more social punishment and higher internalized homophobia (Barrientos et al., 2017; Mann et al., 2019; Bartram, 2021). In the United Kingdom, Mann et al. (2019) found that homosexual and bisexual people report lower life satisfaction than heterosexuals, but these distinctions are heterogeneous and depend also on gender. Studies with samples from the United Kingdom and Australia (Powdthavee and Wooden, 2015; Mann et al., 2019; Bartram, 2021) also highlight two under-researched sexual orientation groups who consistently show lower life satisfaction, due to distinct minority stressors: Bisexuals and those who identify as “other” or “prefer not to say” (i.e., to disclose their sexual orientation), the latter reportedly not being LGBQ, but also not identifying as heterosexual.

Non-heterosexual individuals experience, on average, worse physical and mental health than heterosexuals (Przedworski et al., 2015; Bränström et al., 2016). The minority stress model suggests that heteronormativity leads to stigmatization and discrimination of non-heterosexual people and shows that, in turn, this mistreatment can have adverse effects on health and well-being (Hardy, 2019; Mann et al., 2019; Bible, 2020), and on self-perceived health (Powdthavee and Wooden, 2015). Studies have linked heteronormativity to sexual health in women (Bible, 2020) and psychological functioning in heterosexual and LGBQ women (Habarth et al., 2019b). This second study highlights the importance of accounting for the distinct effects of Essentialism and Normative behavior in health-related measures.

Besides health, one of the most relevant protective factors of well-being, particularly for university students, is the social support perceived from different sources (Zimet et al., 1988; Schnettler et al., 2015; Barrera-Herrera and Vinet, 2017; Orellana et al., 2022). Perceived social support involves being cared for by others and feeling esteemed and valued as part of a social network that entails reciprocal assistance and obligation (Hardy, 2019). The main support sources for university students include family (Schnettler et al., 2017b; Barrera-Herrera et al., 2019), friends (Amati et al., 2018), and other significant persons such as teachers (Seal, 2019; López-Angulo et al., 2020), online social networks (Craig et al., 2021), among others. The COVID-19 pandemic has altered the quality and access that students have to these sources, however. Confinement measures have enforced a physical and social distance that has kept individuals isolated from important social relations, increasing their vulnerability in terms of well-being and mental health (Barrientos et al., 2021).

Social support has been positively linked to several well-being variables including life satisfaction (Domínguez-Fuentes et al., 2012; Schnettler et al., 2015, 2018) and mental health (McDermott et al., 2021). Sexual orientation also plays a role in the nature and effects of social support. In a study of LGBTIQ+ well-being profiles, Hardy (2019) reported that the impact of social support depends on whether the focus is general support (e.g., increased life satisfaction), or it relates specifically to the LGBTIQ+ identity (e.g., decreased internalized sexual prejudice). Moreover, different sources of social support can make distinct contributions to the individual’s well-being when accounting for sexual orientation. A previous study with Chilean university students, conducted during the COVID-19 quarantine period, showed that heterosexuals reported higher family support than lesbians, gays, and bisexuals, but the latter group had higher support from friends and other relevant people (Orellana et al., 2022).

Heteronormativity also plays a role in how individuals engage with different social support sources. Family support is essential for Chilean university students’ development (Schnettler et al., 2015, 2018; Barrera-Herrera and Vinet, 2017), but family relationships are a point of contention for LGBTQI+ people, particularly youth (McDermott et al., 2021). The family is the primary site where heteronormativity is produced and reinforced, fusing together gender, sexual, and family ideologies (Goldberg et al., 2017). Heteronormativity can turn family relationships oppressive and hostile, as families can conduct heteronormative surveillance; LGBTQI+ individuals juggle the need for autonomy and authenticity and the need to stay with their family for belonging and safety (Barrientos et al., 2021; McDermott et al., 2021). However, LGBTQI+ people may also align with heteronormative ideals about family (Pollitt et al., 2021), or they may downplay their sexuality, even as adults, to avoid disrupting family harmony (Goldberg et al., 2017). In contrast with the obligations of family, individuals can freely choose their friendship and other relevant social networks. However, the nature of same-gender and cross-gender friendships and acquaintances can still be conditioned by heteronormativity, given its essentialist assumption of ever-present sexual tension between men and women (Gillespie et al., 2015); the prioritization of coupledom over other emotional bonds (Cronin, 2015); and, particularly for men, because traditional masculinity ideals can preclude them from forming emotional bonds with other men (Ríos-González et al., 2021).

Against this background, the aim of this study was to distinguish heteronormativity profiles of university students, based on Essentialism and Normative behavior. A second aim was to characterize these profiles by sociodemographic characteristics (gender, sexual orientation, faculty, and area of residence), and by their association with well-being variables, namely, life satisfaction, self-perceived physical and mental health, and perceived social support from family, friends, and relevant others, in the context of the COVID-19 pandemic.

## Materials and methods

### Participants

The sample comprised 552 university students in Temuco, Chile, who responded to an online questionnaire. Inclusion criteria were to be over 18 years old and to attend university in Temuco. Although power analysis is not necessary for Latent Profile Analysis (LPA), given the complexity of the parameter values involved, a systematic review on this subject (Spurka et al., 2020) suggests that a sample size of 500 cases allows for a sufficiently accurate identification of the correct number of latent profiles.

Table 1 displays the sociodemographic characteristics of the sample. The mean age of participants was 20.9 years. Most participants were women (74.8%), followed by men (20.9%) and non-binary/fluid (4.3%). Regarding sexual orientation, 45.8% of students were heterosexual, 31.5% bisexual, 10.5% gay or lesbian, 6.5% other orientation (i.e., pansexual, asexual and others grouped here as queer), and a remaining 5.6% preferred not to disclose their sexual orientation. Participants’ gender identity (i.e., cisgender or transgender) was not part of the analysis, but the questionnaire included a question to ask whether the gender reported (woman, men, or non-binary) coincided with the gender assigned at birth (if yes = participant is cisgender, if no = participant is transgender, see Brandelli et al., 2022; by this definition, non-binary genders are categorized under the transgender spectrum). This two-fold distinction was relevant to identify heterosexual transgender students, but all participants who identified as transgender in our sample also identified as non-heterosexual. Lastly, most students reported living in an urban area (78.8%) versus those who lived in a rural area (21.2%), and most belonged to faculties of Health Sciences (26.4%), Social Sciences and Humanities (18.8%), Education (13.8%), and Engineering and Computer Sciences (13.6%).

**Table 1:** Sociodemographic characteristics of the sample.

Variable		%
Age [*M* (SD)]		20.98 (2.82)
Gender	Male	20.8
	Female	74.8
Non-binary	4.3
Sexual orientation	Heterosexual	45.8
	Gay/lesbian	10.5
	Bisexual	31.5
	Other	6.5
	Prefer not to say	5.6
Living with parents	All year round	72.1
	During weekends/holidays	13.4
	Independent from parents	14.5
Area of residence	Urban	78.8
	Rural	21.2
Faculty	Health sciences	26.4
	Social sciences and humanities	18.8
	Legal, economic, and business sciences	9.1
	Education	13.8
	Engineering and computing sciences	13.6
	Agricultural and forestry sciences	3.3
	Architecture, arts and design	7.2
	Other	7.8

### Instruments

#### Sociodemographic questions

This section included questions about participants’ age; gender: men, woman, non-binary; whether this gender coincided with the one assigned at birth (see Participants); sexual orientation: heterosexual, lesbian/gay, bisexual, other (with open-ended question to specify), and prefer not to say; area of residence: urban, rural; and faculty.

#### Heteronormative attitudes and beliefs scale

Habarth (2014) proposed this 16-item scale to operationalize heteronormativity using two dimensions: Essential sex and gender (Essentialism) and Normative behavior. Sample items for each dimension are, respectively, *All people are either male or female* and *In intimate relationships, people should act only according to what is traditionally expected of their gender*. Likert response options range from 1 = Strongly disagree to 7 = Strongly agree. Habarth (2014) reported reliability coefficients of *α* = 0.92 for the Essential sex and gender subscale and *α* = 0.78 for the Normative behavior subscale. We used a shorter, 8-item version of the HABS (HABS-8), translated to Spanish and validated in a sample of Chilean university students, and with each dimension composed of four items (Alarcón et al., manuscript under review). This validation study reported *α* = 0.78 for the whole scale, *α* = 0.73 for the Essential sex and gender subscale, and *α* = 0.77 for the Normative behavior one.

#### Satisfaction with life scale

Diener et al. (1985) proposed this scale, which is composed by five items that evaluate individuals’ global cognitive evaluations of their own life. A sample item is: *In most ways my life is close to my ideal*. Likert response options range from 1 = Completely disagree to 6 = Completely agree. Research using the SWLS in Chilean university simples report Cronbach’s Alpha values ranging from 0.87 to 0.89 (Schnettler et al., 2018). In this study, reliability was *α* = 0.85.

#### Health-related quality of life index (HRQOL-4)

Hennessy et al. (1994) developed this instrument consisting of four items that explore individuals’ overall self-perception of health, recent physical and mental health problems (number of days with illness or discomfort experienced in the last 30 days), and limitations on daily activity due to health issues. We used two of these four items that explored the number of days in which participants experienced either physical or mental health problems in the last 30 days at the time of responding the questionnaire. We used the Spanish version of the HRQOL-4 applied by Schnettler et al. (2017a).

#### Multidimensional scale of perceived social support

Zimet et al. (1988) developed this 12-item scale that measures individuals’ perceived support from family, friends, and other relevant persons. Each of these three dimensions also represent a subscale. Sample items are: *I can talk about my problems with my family*; *my friends really try to help me*; *there is an important person in my life who cares about my feelings*. Likert response options range from 1 = Completely disagree to 7 = Completely agree. Research with Chilean university samples have reported *α* = 0.80 for the whole scale (Orellana et al., 2022). In this study, Cronbach’s alpha values were *α* = 0.89, *α* = 0.92, and *α* = 0.85 for family, friends, and other relevant persons, respectively.

### Procedure

The invitation to participate in this study was distributed through four universities in the city of Temuco and through local student and LGBTIQ+ groups. This invitation included a link to the questionnaire. The first page of this questionnaire displayed the informed consent form (also available for download), which explained the objectives of the study, the inclusion criteria, the voluntary nature of participation, and the anonymous and confidential treatment of the data. Participants were asked to check a box to confirm their participation. This questionnaire was distributed between July and August 2021. Response times ranged between 10 and 15 min. Prior to this procedure, we conducted a pilot test with 24 students who met the inclusion criteria.

This study belongs to a larger research project on sexual orientation and life satisfaction in Chilean university students (ANID – Proyecto Fondecyt Postdoctoral 3210003). This research was approved by the Ethics Committee of Universidad de La Frontera.

### Data analysis

The online questionnaire was hosted on the QuestionPro platform. We analyzed the data using the Statistical Package for Social Sciences (IBM SPSS), v. 26, and we established the heteronormativity profiles using LatentGold v. 5.1 (Statistical Innovations Inc.). We first revised the database to remove incomplete questionnaires and those which did not fulfill the inclusion criteria. We then calculated frequencies and descriptive analysis, overall scores, and Cronbach’s Alpha to examine the reliability of the measures. Score averages and statistical differences by gender and sexual orientation are presented in Supplementary material.

We followed a two-step process to identify heteronormativity profiles based on Essentialism and Normative behavior, the two dimensions of heteronormativity according to Habarth (2014). The first step was to group participants based on their Essentialism and Normative behavior scores. We conducted a latent profile analysis (LPA) for continuous variables to estimate the number of profiles for students, and calculated z-scores for each heteronormativity dimension. We used the Bayesian Information Criterion (BIC) and Consistent Akaike’s Information Criterion (CAIC) values to choose the most fitting solution using gender and sexual orientation as covariates. For these values, lower scores indicate a better model fit.

For the second step in this analysis, we characterized the resulting heteronormativity profiles based on statistical differences in all variables concurrent with these scores. To describe characteristics associated with these profiles, we used Pearson’s Chi^2^ test for discrete variables, and analysis of variance (ANOVA) for continuous variables. We used Levene’s statistic to identify homogeneous and non-homogenous variances in the continuous variables. These variables showed non-homogeneous variances, and thus, we used Dunnett’s T3 Multiple Comparisons test (*p* < 0.001).

## Results

We conducted a LPA to distinguish profiles of heteronormativity in university students. This analysis resulted in an initial run of 1–15 clusters based on the z-scores from Essentialism and Normative behavior (Table 2). The four-cluster model showed the best fit with the lowest BIC and CAIC values (Vermunt and Magidson, 2002). Moreover, in this four-profile solution, the z-scores of the two heteronormativity dimensions made a significant contribution to the overall model, according to the robust Wald statistics and R^2^ values (Table 3).

**Table 2:** Summary of latent profile cluster models.

Model	LL	BIC (LL)	CAIC (LL)	Npar	Classification error
1-cluster	−1565.5072	3156.2687	3160.2687	4	0.000
2-cluster	−1257.7114	2610.1259	2625.1259	15	0.0619
3-cluster	−1095.0183	2354.1889	2380.1889	26	0.0928
4-cluster	−993.4946	**2220.5904**	**2257.5904**	37	0.1175
5-cluster	−959.1077	2221.2657	2269.2657	48	0.1206
6-cluster	−929.1245	2230.7483	2289.7483	59	0.1476
7-cluster	−901.0498	2244.0480	2314.0480	70	0.1381
8-cluster	−881.6162	2274.6298	2355.6298	81	0.1364
9-cluster	−849.6166	2280.0796	2372.0796	92	0.1517
10-cluster	−827.4649	2305.2252	2408.2252	103	0.1242
11-cluster	−799.5098	2318.7641	2432.7641	114	0.1545
12-cluster	−773.7313	2336.6560	2461.6560	125	0.1367
13-cluster	−748.6027	2355.8480	2491.8480	136	0.1274
14-cluster	−752.4883	2433.0681	2580.0681	147	0.1410
15-cluster	−742.0441	2418.6287	2639.6287	158	0.1403

LL, Log-likelihood; BIC (LL), Bayesian information criterion base on the log-likelihood; CAIC (LL), Consistent Akaike’s Information Criterion; Npar, Number of parameters.Values in bold indicate model with the best fit.

**Table 3:** Significance of the indicators for the profiles.

	Robust Wald statistics	*p* Value	*R* ^2^
Essentialism	482.9142	2.4e-104	0.5468
Normative behavior	457.1661	9.1e-99	0.5583

**Figure 1:**
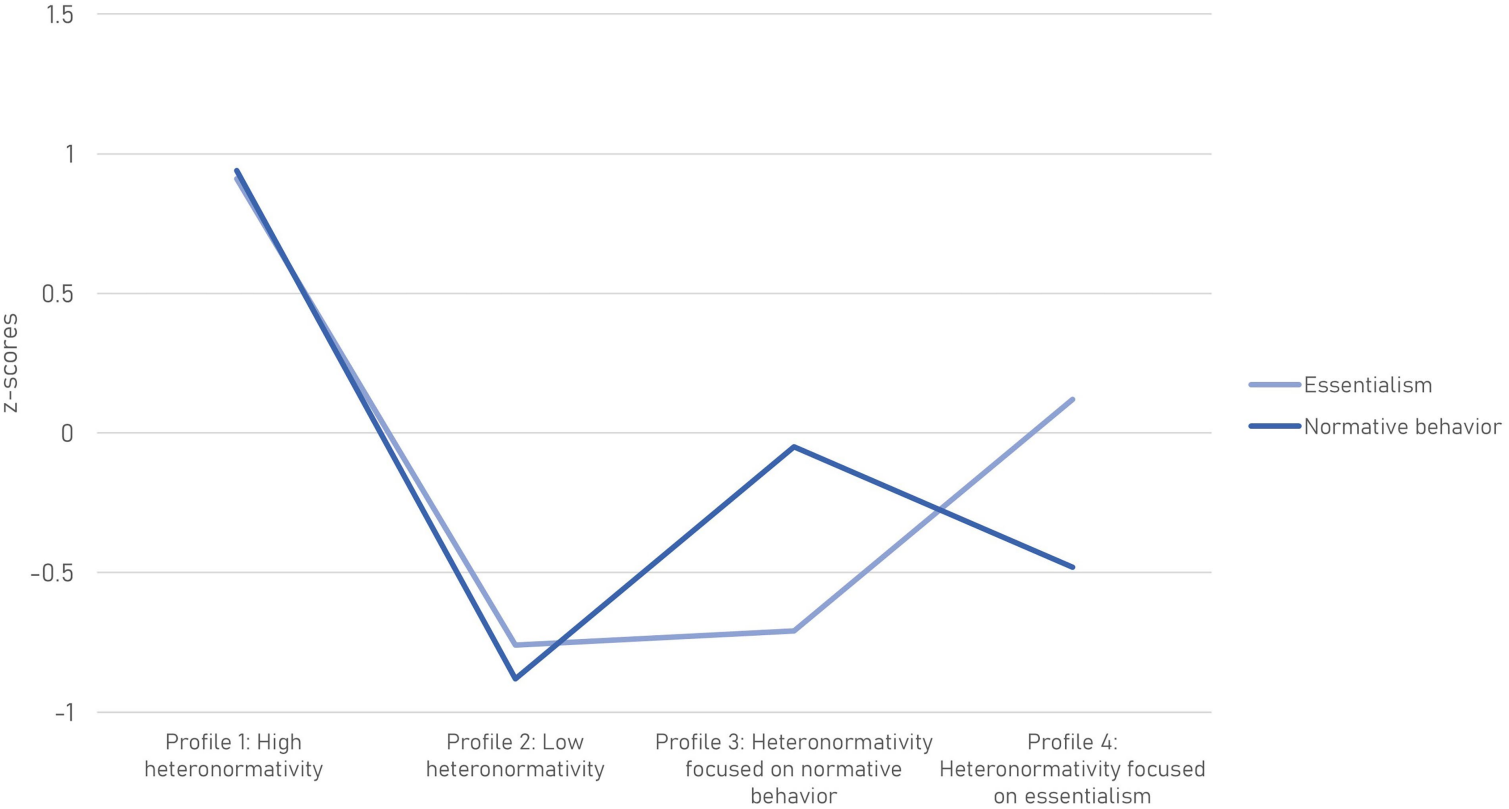
Heteronormativity profiles based on the scores of Essentialism and Normative behavior, the two dimensions of heteronormativity according to Habarth (2014). Differences in each dimension for each profile were *p* < 0.001.

The profiles differed in Essentialism (*F* = 252.143, *p* < 0.001) and Normative behavior (*F* = 279.305, *p* < 0.001), as shown in Figure 1. Students in these profiles also differed by number of days in which they experienced mental health issues (*p* ≤ 0.001), by perceived social support from family, friends, and other relevant persons (*p* ≤ 0.01), and by life satisfaction (*p* = 0.002). Table 4 displays these scores. The profiles did not significantly differ in the number of days with physical health issues (*p* = 0.619). For sociodemographic characteristics, the profiles differed in gender, sexual orientation, area of residence, and faculty (Table 5). The four profiles are described below.

**Table 4:** Average scores (*z*-scores) by profile for perceived social support, number of days with mental health issues in the last month, and life satisfaction.

	Profile 1a (34.85%)	Profile 2b (25.59%)	Profile 3c (20.42%)	Profile 4d (19.14%)	*F*	*p* Value
Family support	14.48 a	14.15 ab	13.00 b	13.13 b	4.79	0.003
Friends support	14.73 b	16.30 a	15.35 ab	15.18 ab	4.38	0.005
Others support	15.46 ab	16.35 a	15.03 b	14.79 b	3.90	0.009
Number of days with mental health issues	15.06 b	17.34 ab	17.25 ab	19.43 a	4.29	0.005
Life satisfaction	18.82 a	17.68 ab	17.26 ab	16.32 b	5.00	0.002

Capital letters on each row indicate significant differences according to Dunnett’s T3 multiple comparisons test. a, High heteronormativity; b, Low heteronormativity; c, Heteronormativity focused on normative behavior; d, Heteronormativity focused on essentialism.

**Table 5:** Sociodemographic characteristics (%) with significant differences by profile.

Variable		Profile 1a	Profile 2b	Profile 3c	Profile 4d	*p* Value
Gender	Male	**35.7**	*12.8*	20.0	*6.8*	<0.001
	Female	*64.3*	79.9	73.9	**87.4**
	Non-binary	*0.0*	**7.4**	6.1	5.8	
Sexual orientation	Heterosexual	**83.8**	*21.5*	*13.0*	49.5	<0.001
	Gay/lesbian	*3.8*	**14.8**	**20.0**	5.8
	Bisexual	*8.6*	**46.3**	**53.0**	27.2
	Other (queer)	*0.5*	**12.1**	9.6	5.8
	Prefer not to say	3.2	5.4	4.3	**11.7**	
Area of residence	Urban	*71.9*	83.2	82.6	80.6	0.042
	Rural	**28.1**	16.2	17.4	19.4
Faculty	Health sciences	30.3	*22.8*	27.0	24.3	<0.001
	Social sciences and humanities	*9.2*	**28.2**	**25.2**	15.5
	Legal, economic, business sciences	11.9	12.1	*4.3*	4.9
	Education	11.9	11.4	10.4	**24.3**
	Engineering, computing sciences	16.8	10.1	14.8	11.7
	Agricultural, forestry sciences	*1.1*	3.4	**7.0**	2.9
	Architecture, arts and design	8.1	6.7	6.1	7.8
	Another	10.8	5.4	5.2	8.7

*p* values obtained from Chi^2^ test. Values in bold represent a statistically high proportion (%) of cases in the profile, indicated by adjusted residuals >2.0. Values in italics represent a statistically low proportion (%) of cases in the profile, indicated by adjusted residuals <−2.0. a, High heteronormativity; b, Low heteronormativity; c, Heteronormativity focused on normative behavior; d, Heteronormativity focused on essentialism.

### Profile 1: High heteronormativity (34.85%)

Students in this profile scored significantly higher than the rest of the profiles in both Essentialism and Normative behavior (*p* < 0.001). These participants also had the highest score in life satisfaction, but this score only differed significantly from Profile 4 (*p* = 0.002). Perceived family support was the highest among the profiles, and this score was significantly higher than for Profiles 3 and 4 (*p* = 0.003). This profile had the lowest score for support from friends, but it only differed statistically from Profile 2 (*p* = 0.005); and it had the second highest score for support from others, but it did not differ from the other three groups. These participants reported fewer days with mental health issues, but this number was only significantly lower than that of Profile 4 (*p* = 0.005). This profile had a statistically higher proportion of men and heterosexual individuals, and a significantly lower proportion of women and non-binary people, and individuals who were LGBQ (*p* < 0.001). This profile had both a high proportion of students living in rural areas and a low proportion of students in urban areas (*p* = 0.042). Lastly, this profile had a significantly lower proportion of students from faculties of Social Sciences and Humanities and Agricultural and Forestry Sciences (*p* < 0.001).

### Profile 2: Low heteronormativity (25.59%)

Participants in this profile had a low score in Essentialism, significantly lower than those of Profile 1 and Profile 4 (*p* < 0.001). Their Normative behavior score was significantly the lowest of all Profiles (*p* < 0.001). Life satisfaction and family support scores in this profile did not significantly differ from the other three profiles. Perceived support from friends was the highest, but it was only significantly higher than that of Profile 1 (*p* = 0.005), while support from others was the highest, and significantly higher than for Profiles 3 and 4 (*p* = 0.009). Participants in this profile did not differ significantly from the other three in terms of number of days with mental health issues. This profile had a significantly high proportion of non-binary students and a significantly low proportion of men (*p* < 0.001); it also had the highest proportion of LGBQ students. A significantly high proportion of students were from faculties of Social Sciences and Humanities (*p* < 0.001).

### Profile 3: Heteronormativity focused on normative behavior (20.42%)

Students in this profile had the lowest score for Essentialism, significantly lower than that Profiles 1 and 4 (*p* < 0.001). Normative behavior was significantly lower than in Profile 1, and significantly higher than in Profiles 2 and 4 (*p* < 0.001). These students had the lowest score for perceived family support, but it was only significantly lower than Profile 1 (*p* = 0.003). This profile did not differ from the other three in terms of support from friends but reported significantly lower support from others compared to Profile 2 (*p* = 0.009). Both life satisfaction scores and number of days with mental health issues were statistically similar to those from the other three profiles. In terms of sexual orientation, it had a significantly low proportion of heterosexual students, and a high proportion of gay/lesbian and bisexual students (*p* < 0.001). There was a high proportion of students from faculties of Social Sciences and Humanities and Agricultural and Forestry, and a low proportion from Legal, economic, and business sciences.

### Profile 4: Heteronormativity focused on essentialism (19.14%)

This profile had a mid-high score in Essentialism, significantly lower than Profile 1 and significantly higher than Profiles 2 and 3 (*p* < 0.001). Conversely, its Normative behavior score was lower than that of Profiles 1 and 3, and significantly higher than Profile 2 (*p* < 0.001). Participants in this profile had a significantly lower score for family support than Profile 1 (*p* = 0.003). Scores for perceived support from friends did not differ from the other profiles, and scores for social support from others differed significantly only from Profile 2 (*p* = 0.009). This profile also had the lowest score in life satisfaction, and the highest number of days with mental health issues, but in both cases, it only differed significantly from Profile 1 (*p* = 0.002 and *p* = 0.005, respectively). There was a significantly low proportion of men and a high proportion of women (*p* < 0.001), and a significantly high proportion of students who preferred not to report their sexual orientation (*p* < 0.001). This profile had a high proportion of students from faculties of Education.

## Discussion

We examined profiles of heteronormativity, composed by essentialism and normative behavior (Habarth, 2014), and well-being among university students. Using Latent Profile Analysis and mean group comparisons, we examined the within-group association patterns of these two dimensions, sociodemographic characteristics, and well-being variables. We found a four-model solution comprising the profiles of High heteronormativity (Profile 1), Low heteronormativity (Profile 2), Heteronormativity focused on normative behavior (Profile 3), and Heteronormativity focused on essentialism (Profile 4). These findings emerge in the context of the COVID-19 pandemic, which has altered social relationships and life trajectories, affected people’s well-being, and increased the vulnerability of minority groups. These results show that heteronormativity can be found in heterogeneous configurations among students of different sexual orientations and genders, and that these configurations may be linked to life conditions and experiences, such as area of residence, faculty, perceived social support, self-perceived mental health, and life satisfaction.

### Essentialism and normative behavior across gender and sexual orientation

The sample was distributed among two “consistent” heteronormativity profiles and two “mixed” profiles, based on their degree of essentialism and normative behavior (see Figure 1). In the consistent profiles, both dimensions had high or low scores, namely, 34.85% of students showed high heteronormativity, while 25.59% showed low heteronormativity. In the two mixed profiles, these dimensions had diverging scores (one high, the other low), with 20.42% of the sample categorized as having Heteronormativity focused on normative behavior, and the remaining 19.14% as having Heteronormativity focused on essentialism (19.14%). These findings support the proposition that Essentialism and Normative behavior are two distinct dimensions of heteronormativity (Alarcón et al., manuscript under review; Habarth, 2014; Scandurra et al., 2021), one related to essentialist binary beliefs about sex and gender, and the other related to attitudes toward the expected roles and behaviors of men and women as individuals and in relationships.

Profiles 1 and 2 displayed the consistency of association between Essentialism and Normative, as two components of a larger construct. Moreover, the sociodemographic characteristics statistically represented in these two profiles are attributes that previous research has associated with heteronormativity. Profile 1, High heteronormativity, was significantly composed by students who were men and heterosexual, in keeping with findings that men have higher heteronormativity than women (Habarth, 2014; Habarth et al., 2019a; Scandurra et al., 2021). This result is expected because heteronormative beliefs and behaviors sustain a social hierarchy that is most protected by heterosexual men (Corlett et al., 2022), because it entails a position of power and advantage over other groups (Farvid, 2015). Additionally, heteronormativity encompasses masculinity norms (Ray and Parkhill, 2021), to which men must rigidly adhere to benefit from this social hierarchy. On the other hand, Profile 2, Low heteronormativity, comprised students who reported both low Essentialism and Normative behavior. This profile also had a significant proportion of students who were lesbian, gay, bisexual or queer (LGBQ) and of a non-binary gender. Individuals with these characteristics are the most at risk of experiencing minority stress derived from heteronormativity (Mann et al., 2019), as the markers of their sexual and gender identities (e.g., patterns of attraction, gender expression) stand against binary and essentialist expectations about how “men and women” are and how they behave, individually and toward one another.

The other two profiles, Profiles 3 and 4, are mixed because they show different configurations of Essentialism and Normative behavior, supporting evidence that these two dimensions of heteronormativity are correlated but distinct (Alarcón et al., manuscript under review; Habarth, 2014; Scandurra et al., 2021). Profile 3, Heteronormativity focused on normative behavior, was significantly comprised by lesbian, gay and bisexual (LGB) students, with no significant proportions by gender. The finding that LGB students adhere to heteronormative behavior without significantly endorsing essentialist beliefs may be explained by a contextual factor and by cognitive-cultural schemas. We address the contextual factor, the confinement measures during the COVID-19 pandemic, in our discussion about family support. On the other hand, a cognitive-cultural schema guides compliance with heteronormativity through artifacts and activities, leading people to behave following a ritualized action or a normative expectation (Corlett et al., 2022). LGB individuals may thus have positive attitudes toward Normative behavior because these norms are expected, appropriate, or taken for granted. Moreover, compliance with normative behavior may be an identity management strategy to avoid disclosing their sexual orientation (or gender identity) to others; disclosure is both a proximal stressor and a protective factor in the minority stress model (Meyer, 2003), because “doing sexuality” is exposing oneself to risk (Goldberg et al., 2017). Hence, Profile 3 shows that LGB people are not automatically “beyond heteronormativity” (Beltrán y Puga, 2012). These individuals challenge aspects of heteronormativity that question the foundations of their identity (e.g., sexual and gender essentialism), but they can also maintain –due to internalization or for their safety– beliefs and behaviors that reinforce the appropriateness of normative sexual and gender expressions and partnership/family configurations (Goldberg et al., 2017; Pollitt et al., 2021).

The second mixed profile was Profile 4, Heteronormativity focused on essentialism, which comprised students who had both a distinctly high score in Essentialism and a low score in Normative behavior, compared to the other three profiles. This profile had a significant proportion of women and of students who preferred not to report their sexual orientation. Pollitt et al. (2021) posit that heteronormativity can be indeed reinforced by upholding traditional beliefs about gender, sexuality, and related constructs, such as family. Students in this profile may classify sexuality, and specifically heterosexuality, as a natural quality that precedes social life (Maldonado-Ramírez, 2015). Given its proportions by gender, this profile appears to comprise a disadvantaged group. Nevertheless, for people such as those in Profile 4, the question remains why the acceptance of heteronormative behavior does not significantly manifest alongside these essentialist beliefs. A possible explanation is that women are highly exposed to heteronormative messages that stigmatize them and their sexuality (Bible, 2020), and resistance to this stigma (Seal, 2019) may take the form of challenging gendered expectations through non-normative behavior. A second explanation may be related to the current measure of Normative behavior from the Heteronormative Attitudes and Belief Scale (HABS). Habarth et al. (2019b) have hypothesized that it may be not assess attitudes that are relevant to women, particularly heterosexual ones. Future studies should address this possibility and expand on this measure.

One last notable characteristics of Profile 4 is the significant proportion of students who preferred not to disclose their sexuality. Other researchers have encouraged to observe this group (Powdthavee and Wooden, 2015; Mann et al., 2019; Bartram, 2021). These are individuals who do not identify as heterosexual nor as LGBQ but report lower life satisfaction than heterosexuals. Thus, they may be experiencing systematically different protective and risk factors related to their sexual orientation and well-being.

We identified two other sociodemographic differences in these profiles. The first one is area of residence. Profile 1 had a significantly higher proportion of students from rural areas, which have been characterized as contexts of lower acceptance of non-normative sexual and gender identities, compared to urban areas (Barrientos-Delgado et al., 2014; Giano et al., 2020). A second sociodemographic difference between profiles was faculty. This finding is in keeping with the notion that occupational sectors and industries are gendered (Corlett et al., 2022). Feminized fields were significantly represented in the three profiles with high proportions of women and non-binary people and non-heterosexuals: Social Sciences and Humanities in Profiles 2, 3, and 4, and Education in Profile 4. Some studies (Maldonado-Ramírez, 2015; Phipps, 2020; Corlett et al., 2022) highlight that masculinized fields (e.g., law enforcement, engineering, certain sports) consider “feminine others” as a threat to be controlled and punished, which maintains a power hierarchy with traditionally masculine heterosexual men at the highest positions. This distinction by faculties in the profiles is relevant to the discussion of how to identify and challenge heteronormativity in higher education (see Seal, 2019). Future research should explore the sexuality and gender norms that are reinforced and those that are questioned among students and staff from different academic fields.

### Heteronormativity and well-being indicators

We characterized heteronormativity profiles with variables that the literature links to subjective well-being: Life satisfaction, social support from family, friends, and relevant others, and a measure of self-perceived physical and mental health (i.e., number of days with physical and mental health problems in the last month). These variables have been previously explored in Chilean university students (Schnettler et al., 2015, 2017b; Barrera-Herrera and Vinet, 2017), and have been assessed by sexual orientation in youth and adult populations (Powdthavee and Wooden, 2015; Bränström et al., 2016; Cooke, 2018; Pachankis and Bränström, 2018; Hardy, 2019). The latter line of research indicates that non-heterosexual people experience lower well-being compared to their heterosexual counterparts, albeit with nuances related to concurrent individual characteristics and life conditions. Our results support these nuances in life satisfaction, social support, and self-perceived health, and highlight the heterogeneity in the association patterns between these variables and the two dimensions of heteronormativity.

For life satisfaction, based on previous findings (Powdthavee and Wooden, 2015; Mann et al., 2019; Bartram, 2021) and on the minority stress model (Meyer, 2003; Meyer et al., 2021), we expected that profiles with significant proportions of non-heterosexual students (LGBQ and those who preferred not to identify) would report lower life satisfaction than profiles with heterosexual students. On the contrary, the profiles with the highest proportions of LGBQ students, Profiles 2 and 3, were statistically undistinguishable from the other two profiles in terms of life satisfaction. Studies with adults from Australia and the United Kingdom (Powdthavee and Wooden, 2015), and from other European countries (Pachankis and Bränström, 2018), show that non-heterosexual individuals experience economic, social, and personal factors that explain their lower life satisfaction compared to heterosexual people. In our sample, there may be factors related to culture (developed versus developing countries), life period (adulthood versus emerging adulthood/attending university), and context (COVID-19 pandemic) that can help explain the similarities in life satisfaction by sexual orientation among students in these profiles. Another study conducted during the pandemic with Chilean university students found no differences in life satisfaction between heterosexual and LGB students (Orellana et al., 2022). These findings suggest that, as Chilean university students have shown a mid-to-low baseline of life satisfaction (Schnettler et al., 2015, 2017b), there may be intermediate protective factors against minority stressors for non-heterosexual students. Hence, their life satisfaction levels are like those of their heterosexual peers.

The significant difference in life satisfaction levels was instead found in Profiles 1 and 4. These profiles were composed by a significant proportion of, respectively, men and heterosexuals, and women and those who did not disclose their sexual orientation. This result suggests the coexistence of high life satisfaction and high heteronormativity for –mostly– men and heterosexuals, and the coexistence of high essentialism and low life satisfaction for women and those who do not disclose their sexual orientation.

In terms of undisclosed sexual orientation, our findings coincide with those by Powdthavee and Wooden (2015). These authors found that United Kingdom and Australian heterosexuals reported higher life satisfaction than those who “preferred not to say.” Moreover, Habarth et al. (2019b) found that non-heterosexual women with stronger essentialist beliefs report lower well-being than heterosexual women, and this may be the case for non-heterosexual women in Profile 4. Nevertheless, the distinctions in life satisfaction here appear to be more prominent by gender rather than by sexual orientation, between a group significantly composed by men who adhere to heteronormativity (Profile 1) and a group significantly composed by women who adhere to essentialist sex and gender beliefs (Profile 4). For the latter group, essentialism may be contributing to perpetuate gendered beliefs that place women in a submissive status compared to men (Farvid, 2015). Essentialism may thus be linked to an increased risk of experiencing gender-based victimization, and this in turn can have a negative impact on their life satisfaction.

Another well-being variable that we included was perceived social support from family, friends, and relevant others. Previous research underscores that family support is fundamental for the development and subjective well-being of university students and emergent adults in general (Schnettler et al., 2015, 2017b; Barrera-Herrera and Vinet, 2017). Other studies on the topic that include sexual orientation show that heterosexual and non-heterosexual people engage differently with their families and receive differential benefits from their support (Goldberg et al., 2017; Hardy, 2019; McDermott et al., 2021). Our findings contribute to this body of research by associating levels of heteronormativity with degrees of family support, as university students with High heteronormativity (Profile 1) received higher family support than those with mixed heteronormativity (Profiles 3 and 4). Based on studies with LGBTQI+ youth and their family relations (Barrientos et al., 2021; McDermott et al., 2021), we hypothesize that university students with heteronormative attitudes will face less conflict with their families and will continue receiving emotional and material resources (e.g., shelter, encouragement, economic support).

For non-heterosexual young adults, the family is both a protective and a risk factor for their well-being. The literature is consistent in showing that these individuals receive less family support than their heterosexual peers (Orellana et al., 2022). However, in our study, students with Low heteronormativity (Profile 2), significantly composed by LGBQ and non-binary students, were statistically undistinguishable from the other three profiles in terms of family support. We propose two tentative explanations for this result. First, these students may experience an overall supportive family environment, with low heteronormativity as an associated condition, whether as an antecedent or a consequence. Second, these students exercise their agency and do an extensive emotion work to maintain harmonious family relationships, negotiating between heteronormative family discourses and their own sexual and/or gender identities (McDermott et al., 2021). This negotiation, which can include total or partial concealment of their non-normative identities, can be vital for LGBQ and non-binary students to maintain the support that their family provides.

Social support from friends also differed significantly between those with High heteronormativity and Low heteronormativity, while the mixed heteronormativity profiles reported statistically similar levels. Based on this result, we suggest that higher heteronormativity is associated with smaller friendship networks or support. Under a heteronormative logic, only certain types of relationships can occur between men and women (i.e., sexual and romantic relationships), hence cross-gender friendships are avoided or kept to a minimum to prevent sexual tension (Gillespie et al., 2015). Individuals may be prevented, and/or prevent their partners, from having friends of the “opposite gender” as heteronormative conceptions of cross-gender relationships are framed within sexual and romantic attraction. Furthermore, traditional masculinity roles limits men’s possibilities to establish emotional relationships, particularly with other men (Ríos-González et al., 2021). Of note, however, individuals who are, or partner with, people of same-gender or multiple-gender attraction, can also have their same-gender and cross-gender friendships influenced by heteronormative expectations (Gillespie et al., 2015; Seal, 2019). Overall, heteronormativity can lead to deprioritize friendships, and other intimacy and emotional bonds outside the couple, and even frame these relations as a threat to the couple (Cronin, 2015). Previous studies have linked friendship relations to life satisfaction (Amati et al., 2018), and the role of heteronormativity in these links should also be explored in future research.

Lastly, for the third type of support examined in this study, those with Low heteronormativity also reported higher support from others, compared to people with mixed heteronormativity (Profiles 3 and 4). On the other hand, students with High heteronormativity were undistinguishable from the rest of the profiles regarding support from others. This finding may support the previous idea that individuals with Low heteronormativity are able to establish more emotional bonds outside traditional sources of support (friends, family) than those with mixed heteronormativity. However, there is still the question of why individuals with high heteronormativity report similar levels of this type of support, if they would be more constrained by gendered norms around relationships. The answer may lie in the type of relevant others that individuals such as those in Profile 1 (men, heterosexuals) and Profile 2 (LGBQ, non-binary) seek and the type of support these others provide. This is a question to explore in future research.

The last well-being indicator that we examined was self-perceived health, operationalized as the number of days in which participants experienced physical and mental health issues in the last month at the time of responding the questionnaire. There were no significant differences among profiles in the number of days with physical difficulties. For mental health difficulties, we found a high number for all groups, reporting between 15 to 19 out of 30 days with these difficulties. This is a concerning but unsurprising finding, considering the increase in mental health issues during the COVID-19 pandemic in both the general population and vulnerable groups (Orellana and Orellana, 2020; Barrientos et al., 2021). In our profiles, as it occurred with life satisfaction, we observed statistical differences between those with High heteronormativity (Profile 1) and those holding essentialist beliefs (Profile 4), with significantly fewer and more days of mental health issues, respectively. The composition of Profiles 1 and 4 again suggest that differences in self-perceived mental health relate to heteronormativity not only in terms of sexual orientation, but also gender. Habarth et al. (2019b) showed that heterosexual women with more strongly essentialist beliefs also reported lower depression. Our results expand on this phenomenon by showing that high heteronormativity is accompanied with –comparatively– better self-perceived mental health, particularly for those who fall within the acceptable boundaries of these norms (men, heterosexuals).

Profiles 2 and 3, significantly composed of LGBQ and non-binary students, did not differ from the rest of the profiles in terms of self-perceived mental health. Most studies on health by sexual orientation in adults from developed countries indicate that non-heterosexual individuals experience more mental health difficulties than heterosexual ones, with more marked distinctions between bisexual and heterosexual people (Meyer, 2003; Powdthavee and Wooden, 2015; Przedworski et al., 2015; Perales, 2019). Moreover, Habarth et al. (2019b) found that non-heterosexual women who endorsed normative behavior –characteristics found in Profile 3– reported lower psychological well-being (i.e., autonomy, growth, sense of purpose). Our findings do not support this evidence, but they align with a study with Portuguese high school students which showed that LGB and heterosexual participants had similar mental health levels (Fonseca de Freitas et al., 2021). Hence, besides heteronormative attitudes, factors related to culture, life period, and the COVID-19 pandemic may be operating in these mixed results regarding mental health.

### Limitations and future research

This study is not without limitations. First, our sample was self-selected and non-probabilistic, from a region in Southern Chile characterized by a conservative culture, compared to other regions of the country. We cannot generalize these findings to the national population of university students, nor at a larger level. A second limitation is that responses may have been driven by conditions related to the COVID-19 pandemic (e.g., confinement in the family home, suspension of in-person classes and activities in campus). These conditions were not assessed in this study, and our data does not provide information to infer the impact of the pandemic in these responses compared to pre-pandemic times. Another limitation is that we did not differentiate students in our profiles by gender identity, that is, between cisgender and transgender participants. The latter category can include non-binary identities, but these were identified as a gender category rather than as gender identity (see Participants). We have highlighted gender identity processes alongside sexual orientation in this paper whenever applicable (e.g., disclosure), and we established that all transgender and non-binary participants in our sample were also non-heterosexual. Nevertheless, heteronormativity also encompasses prejudice and beliefs about transgender and non-binary people to privilege a cisgender worldview (i.e., cisgenderism). Distinguishing gender identity in these profiles would have provided a richer understanding of how transgender and non-binary students experience heteronormativity and how it relates to their well-being.

Another limitation is that the measure of self-perceived mental health consisted of only one item, and its response depended on the person’s interpretation of what mental health entails. This is a limitation particularly regarding male participants, who may be more constrained by traditional masculinity expectations to present themselves—intentionally or unintentionally—as “mentally strong,” or to fail to conceptualize certain experiences as part of the mental health continuum (e.g., see Seal, 2019 on masculinity as an isolating experience). Nevertheless, the number of days reported by this group was still concerning (15 out of 30 days) and it requires further attention as the pandemic progresses. On its part, the measure of heteronormativity might also be prone to social desirability bias and not fully encompass dimensions that are relevant for certain participants (e.g., heterosexual women, Habarth et al., 2019b). Thus, this scale may not capture heteronormativity aspects that may be more strongly associated with well-being.

Future research with university students should include samples with probabilistic distributions across gender, faculty, and area of residence, to test the number and configuration of profiles found here. Future studies should also expand on the measures of well-being, conditions during and after the pandemic, and control for social desirability in the responses to the heteronormativity scale. The manifestations and outcomes of heteronormativity should also be further examined both in relation to discrimination, and in samples of gay, lesbian, bisexual, trans, intersex, and other queer people; we also advise that these groups are examined separately. Heteronormativity is made up by dispositional, attitudinal, relational, and structural assumptions, and belonging to stigmatized groups does not grant immunity from heteronormative beliefs and behaviors. Studies on heteronormativity in LGBQ populations will also benefit from analyzing links between this construct and internalized homophobia, and sexual double standards (i.e., higher internalized homophobia may be associated with higher heteronormativity, and with stronger double standards). Lastly, heteronormativity studies will benefit from including other personal and sociodemographic characteristics (e.g., ethnicity, religion, socioeconomic status) to offer an intersectional approach to how heteronormativity and its two dimensions are experienced by individuals based on their multiple identities and diverse life experiences.

### Research and practical implications

The empirical testing of heteronormativity is a relatively recent endeavor in psychological research, and thus its measurement may not yet encompass all relevant factors of this construct. The first implication from this study for research on heteronormativity is to continue exploring the connection between Habarth’s (2014) construct of heteronormativity and other variables besides sexuality and gender (Seal, 2019). Heteronormativity imposes regulations not only on the attraction, gender and gender expression of the person and their partner (s), but also their age, socioeconomic status, ethnicity, nationality, religion, disabilities, among others (Maldonado-Ramírez, 2015).

Among these variables, family dynamics is perhaps the most immediate issue because the idea of family is indivisible from “doing gender” and “doing sexuality” (Goldberg et al., 2017). The focus on family is of special relevance in Latin American cultures, where social institutions reinforce heteronormativity by priming “opposite but complementary” social roles of men and women in a family unit (see Vergara, 2020). Emergent adults are developing their life trajectory (Nelson, 2020), and heteronormative discourse and behaviors, in both the family (Pollitt et al., 2021) and in higher education (Seal, 2019), can permeate this trajectory. LGBTIQ+ emergent adults can also adhere to heteronormativity, for instance, by adopting heteronormative behaviors to conceal their sexual orientation or gender identity for their safety, or by internalizing assumptions about what constitutes an ideal family and whether it is attainable to them as members of a marginalized group (Pollitt et al., 2021).

There are also valuable research avenues in accounting for the presence of heterosexuals and men in Profiles 2, 3 and 4. Seal, (2019) has underscored that heterosexuality is needed to challenge heteronormativity (e.g., by rendering itself visible). Therefore, identifying further characteristics and experiences of heterosexuals—specially men—with low or mixed heteronormativity can be a gateway to understanding how to increase acceptance of LGBTQI+ people in all life spheres.

This study also has practical implications. Each of the four profiles suggests patterns of heteronormative beliefs that will have a differential impact in students’ well-being. Based on our findings and on previous literature, those students with high heteronormativity are more likely to endorse a hierarchical system of sexual value –an understanding what is normal and what is deviant for sexuality and gender–, and act accordingly to participate in this system (Maldonado-Ramírez, 2015; Wilson, 2022), and stigmatize, harm, and exclude those who threaten it (Ray and Parkhill, 2021; Corlett et al., 2022). Higher education institutions must examine how their discourses and curricula are informed by a heteronormative point of view (see Alarcón et al., manuscript under review; Seal, 2019). Even in progressive environments, these viewpoints can frame non-heterosexual and transgender/non-binary gender identities, at best, as benign deviations from the norm.

Moreover, the patterns of well-being variables linked to heteronormativity can suggest focus points for policies, resources, and services that universities can offer (i.e., health services, student societies) to enhance protective factors for students’ well-being. These resources can be particularly beneficial for students resembling those in profiles with low or mixed heteronormativity, who may also be at a social disadvantage due to their gender or sexual orientation (women and non-binary people, non-heterosexuals). Nevertheless, changes in the social environment have been found to be insufficient to decrease minority stress (Meyer et al., 2021). Therefore, higher education institutions should seek to enhance well-being factors (e.g., increasing support resources for students) alongside cultural and curricular changes regarding sexuality and gender norms (Meyer et al., 2021).

### Conclusion

The four profiles found in this study highlight the need to approach heteronormativity using an intersectional framework. This approach is needed because this construct can manifest in a myriad of ways that depend on the person’s individual characteristics, immediate context, and their social environment. Furthermore, while high heteronormativity appears to coexist with protective factors in our study, the ramifications of heteronormativity are harmful for all people (Seal, 2019). Farvid (2015) states that people who adhere to heteronormativity might show better psychological adjustment, but these beliefs sustain gender stereotypes and power relationships that facilitate discrimination, gender inequality and sexual violence (see Wilson, 2022). Expectations regarding gender and sexual orientation affect those who transgress these norms the most, but rarely any individual will consistently satisfy all these expectations (Habarth, 2014). These profiles suggest research directions to better understand the health and social disadvantages faced by both those who question heteronormativity and those who endorse it.

## Data availability statement

The raw data supporting the conclusions of this article will be made available by the authors, without undue reservation.

## Ethics statement

The studies involving human participants were reviewed and approved by Comité Ético Científico de la Universidad de La Frontera. The patients/participants provided their written informed consent to participate in this study.

## Author contributions

LO and BS conceptualized and designed the study, and performed the statistical analysis. LO and TA collected the data and wrote the first draft of the manuscript. TA organized the database. All authors have read, revised, and approved the final version of the manuscript.

## Funding

Funding for this study was provided by ANID – Proyecto Fondecyt Postdoctoral 3210003.

## Conflict of interest

The authors declare that the research was conducted in the absence of any commercial or financial relationships that could be construed as a potential conflict of interest.

## Publisher’s note

All claims expressed in this article are solely those of the authors and do not necessarily represent those of their affiliated organizations, or those of the publisher, the editors and the reviewers. Any product that may be evaluated in this article, or claim that may be made by its manufacturer, is not guaranteed or endorsed by the publisher.
