# Supplementary material for: Behavior without beliefs: Profiles of heteronormativity and well-being among heterosexual and non-heterosexual university students in Chile
Source: Front Psychol. 2022 Aug 15;13:988054. doi: 10.3389/fpsyg.2022.988054 (PMC9421161; doi:10.3389/fpsyg.2022.988054)
Supplement: Supplementary file 1 [file Table_1.DOCX]

**Behavior without beliefs: Profiles of heteronormativity and well-being among heterosexual and non-heterosexual university students in Chile**

Supplementary Material

Table 1 - Supplementary. Average scores and statistical differences for perceived social support, number of days with mental health issues in the last month, and life satisfaction by gender.

| Variables | Men | | Women | | Non-binary | | F | *p* |
| --- | --- | --- | --- | --- | --- | --- | --- | --- |
|  | M | SD | M | SD | M | SD |  |  |
| Heteronormativity - Essentialism | 13.84 A | 7.02 | 11.13 B | 6.12 | 7.63 c | 4.98 | 13.25 | < .001 |
| Heteronormativity - Normative behavior | 9.40 A | 4.91 | 7.49 B | 3.93 | 5.54 B | 2.35 | 13.51 | < .001 |
| Social support – Family | 13.83 A | 3.87 | 13.96 A | 3.93 | 11.54 B | 4.88 | 4.23 | .015 |
| Social support – Friends | 15.40 A | 4.23 | 15.36 A | 3.95 | 15.33 A | 4.28 | .005 | .995 |
| Social support - Others | 15.25 A | 4.02 | 15.66 AB | 3.90 | 13.54 A | 5.13 | 3.46 | .032 |
| Days with physical health issues | 5.35 A | 8.24 | 6.99 A | 8.64 | 8.38 A | 9.73 | 2.10 | .123 |
| Days with mental health issues | 14.78 A | 10.50 | 17.27 AB | 9.99 | 21.71 B | 10.65 | 5.48 | .004 |
| Life satisfaction | 17.38 A | 5.51 | 17.94 A | 5.45 | 15.50 A | 6.43 | 2.50 | .082 |

*Notes.* Capital letters on each row indicate significant differences according to post-hoc comparison tests.

Table 2 - Supplementary. Average scores and statistical differences for perceived social support, number of days with mental health issues in the last month, and life satisfaction by sexual orientation.

| Variables | Heterosexual | | Gay/lesbian | | Bisexual | | Other | | Prefer not to say | | F | *p* |
| --- | --- | --- | --- | --- | --- | --- | --- | --- | --- | --- | --- | --- |
|  | M | SD | M | SD | M | SD | M | SD | M | SD |  |  |
| Heteronorm. - Essentialism | 14.56 A | 6.47 | 8.59 b | 5.31 | 9.10 b | 5.21 | 8.03 b | 4.97 | 10.26 b | 4.58 | 32.32 | < .001 |
| Heteronorm. - Normative behavior | 9.53 A | 4.69 | 6.43 b | 3.35 | 6.26 b | 2.83 | 5.72 b | 2.39 | 7.32 b | 4.15 | 23.71 | < .001 |
| Social support – Family | 14.60 a | 4.12 | 13.10 ab | 3.50 | 13.11 b | 3.71 | 13.33 ab | 4.10 | 13.55 ab | 4.12 | 4.55 | .001 |
| Social support – Friends | 15.11 ab | 4.29 | 15.52 ab | 3.71 | 15.83 b | 3.55 | 16.39 b | 3.21 | 13.39 a | 4.93 | 3.35 | .010 |
| Social support - Others | 15.79 a | 4.20 | 15.50 a | 3.38 | 15.40 a | 3.88 | 15.03 a | 3.29 | 13.97 a | 4.57 | 1.62 | .166 |
| Days w/ physical health issues | 7.46 a | 9.31 | 5.60 a | 8.09 | 6.17 a | 7.60 | 6.75 a | 7.70 | 5.55 a | 10.03 | 1.03 | .391 |
| Days w/ mental health issues | 16.17 a | 10.6 | 16.91 a | 9.63 | 18.29 a | 9.42 | 19.28 a | 10.54 | 13.10 a | 10.30 | 2.72 | .029 |
| Life satisfaction | 18.49 a | 6.03 | 16.86 ab | 4.89 | 16.95 b | 5.02 | 17.50 ab | 5.11 | 17.58 ab | 4.81 | 2.47 | .043 |

*Notes.* Capital letters on each row indicate significant differences according to post-hoc comparison tests.
